# A systematic review of reviews on the psychometric properties of measures of older persons’ ability to build and maintain social relationships

**DOI:** 10.1093/ageing/afad106

**Published:** 2023-10-30

**Authors:** Pedro Lobo Julião, Óscar Brito Fernandes, Janice P Alves, Jotheeswaran Amuthavalli Thiyagarajan, Christopher Mikton, Theresa Diaz, Sandra Pais

**Affiliations:** Faculty of Medicine and Biomedical Sciences, University of Algarve, Faro, Portugal; Comprehensive Health Research Centre (CHRC), Universidade de Évora, Évora, Portugal; Amsterdam UMC Location University of Amsterdam, Public and Occupational Health, Meibergdreef 9, Amsterdam, The Netherlands; Public Health research institute, Quality of Care, Amsterdam, The Netherlands; Neurology Department, Setúbal Hospital Center, Setúbal, Portugal; School of Health, Polytechnic Institute of Setúbal, Setúbal, Portugal; Ageing and Health Unit, Department of Maternal, Newborn, Child, Adolescent Health and Ageing, World Health Organization, Geneva, Switzerland; Demographic Change and Healthy Ageing Unit, Department of Social Determinants of Health, World Health Organization, Geneva, Switzerland; Epidemiology, Monitoring and Evaluation Unit, Department of Maternal, Newborn, Child, Adolescent Health and Ageing, World Health Organization, Geneva, Switzerland; Faculty of Medicine and Biomedical Sciences, University of Algarve, Faro, Portugal; Comprehensive Health Research Centre (CHRC), Universidade de Évora, Évora, Portugal

**Keywords:** functional capacity, healthy ageing, social relationships, systematic review, older people

## Abstract

**Background:**

Within the scope of the World Health Organisation’s (WHO) world report on ageing and health and how healthy ageing was conceptualised, the WHO has been working with academia towards producing reviews of the psychometric properties of instruments that measure different domains of functional ability. This study aimed to conduct a review of reviews to examine existing and validated instruments measuring the ability of older persons to build and maintain social relationships and to evaluate the psychometric properties of these instruments.

**Methods:**

We searched for studies published in the English, Spanish and Portuguese languages. No restrictions were placed on the year of publication. The following databases were searched: PubMed, Embase, Psyinfo and Cumulated Index to Nursing and Allied Health Literature. Titles and abstracts were screened and selected articles were screened and reviewed independently by two reviewers.

**Results:**

A total of 3,879 records were retrieved, of which 39 records were retrieved for full-text analysis. None of the reviews met the inclusion criteria, thus resulting in an empty review.

**Conclusions:**

Considering the current definition of older persons’ functional ability to build and maintain social relationships, this review did not identify instruments that can measure both constructs simultaneously. We suggest the development of an instrument that simultaneously assesses the ability of older persons to build and maintain relationships.

## Key Points

- Understanding both the ability to build and maintain social relationships in older persons is key.
- There are currently no validated instruments measuring older persons’ ability to build and maintain social relationships.
- A tool is needed to measure the bio-eco-psycho-social factors explaining how older persons build and maintain social relations.

## Introduction

The World Health Organisation (WHO) defined healthy ageing as the process of developing and maintaining the functional ability that enables well-being into old age [1]. In turn, functional ability is defined as the actual or potential capacities that enable a person to perform essential activities of daily living that they value [2]. These capacities can be both physical and mental, and generally can be grouped into five key domains that are relevant to the healthy ageing process, such as the capacities: (i) to meet basic needs (e.g. eating, drinking and sleeping); (ii) to be mobile; (iii) to learn, thrive and make decisions; (iv) to contribute to society and (v) to build and maintain social relationships [1]. This conceptualisation of functional ability highlights the importance of a person’s environment and the quality of the interactions between the individual and the environment. Measuring functional ability becomes increasingly important amongst older people as it can inform about an individual’s quality of life [2]. The scope of this paper is on the domain related to the ability to build and maintain social relationships.

Social relationships of older persons are multidimensional and involve interaction with various social groups, such as their family, intimate relationships and informal and formal relationships [1]. Social relationships of older persons have specific characteristics compared to those of younger adults. For example, older persons tend to have smaller social networks, but the quality of their relationships is often higher. Older persons tend also to rely more on close relationships with family and friends, rather than on a large number of acquaintances. Therefore, loneliness and social isolation (definitions in Supplementary Appendix 1) frequently occur amongst older persons, which are likely to indicate poorer social support, quality of life and overall health. Social support is a broad construct that describes the network of social resources available to support an individual [3]. The notion of social support is an important indicator of social relationships, which builds off robust evidence on how social support impacts loneliness [4]. For example, as people get older, the need of social support generally increases and the more common sources of support are provided by family and close friends [5].

### Conceptualising ability to build and maintain social relationships

The ability to build and maintain social relationships shows a strong relationship with intrinsic capacities (related to a good state of cognitive health), individual characteristics (for example, sex, gender, ethnicity, education and social dysfunctions) and the social environment where older persons interact [1]. The ability to build and maintain social relationships is also closely related to other concepts, such as loneliness, social isolation, social connections, social interactions, social networks, social support and social ties. This web of connections may lead to some overlap amongst these concepts. To mitigate the consequences of this overlap, we clarify the definitions used in this study in Supplementary Appendix 1.

As people grow older, the risk of becoming socially isolated and lonely increases, both of which are negatively associated with mental and physical health [6]. Later in life, the lack of trust in other people has been considered a precondition for loneliness and linked to the reduction of social engagement [7]. Amongst children and young adults, extracurricular activities for life outside school, workplaces and leisure activities generate favourable environments for the creation of new relationships. However, with retirement, significant changes in daily life limit opportunities to meet new people or maintain contact with previous co-workers and peers. These changes have implications in both social roles and networks and are frequently associated with mental health decline or depression [8]. Hence, establishing adequate ways of screening social determinants of health, such as social isolation and loneliness, have been considered crucial [9]. Thus, it is important to accurately measure over time amongst adults the ability to build and maintain relationships in the scope of active and healthy ageing processes.

Only a few instruments seem to be able to measure active ageing as conceptualised by WHO. Yet, there is a wealth of instruments being used in ageing surveys to older persons, although many focus on specific chronic diseases, deficits or other clinical conditions. This study aims to conduct a systematic review of reviews towards identifying existing instruments that can assess the ability of older persons to build and maintain social relationships and to evaluate the psychometric properties of these instruments.

## Methods

A first preliminary database search was conducted to identify studies of interest and test search keywords. However, the results during this process led to a very large number of studies, many of which were outside the scope of this study. To make this study feasible, we then decided to search for systematic reviews relevant to identifying existing instruments measuring the ability of older persons to build and maintain social relationships. Thus, we decided to conduct a systematic review of reviews following the Preferred Reporting Items for Systematic Reviews and Meta-Analyses (PRISMA) guidelines. The terminology and procedures to conduct the review were discussed with WHO colleagues over monthly follow-up meetings (from February to July 2022) for feedback and improvement, and later agreed upon amongst the research team. In September 2022, the preliminary results of this study were discussed in an online meeting of international experts working with WHO on measures of functional ability. The study protocol of this review was registered with the International Prospective Register of Systematic Reviews (PROSPERO) in January 2022.

### Search strategy and selection criteria

The literature search was carried out independently by two researchers (PJ and SP). The databases consulted were PubMed, Embase (Medical database), Psyinfo and the Cumulated Index to Nursing and Allied Health Literature. The constructs of interest were organised around three categories: instrument properties, target population and object of instrument assessment. The search strategy was a combination of subject headings and free-text words, which were adapted for each database as needed and informed by initial exploratory searches (Supplementary Appendix 2). The search was performed between January 2022 and February 2022 and later updated on 5 June 2022. All studies (reviews) retrieved were imported to the open-source application Rayyan [10].

After removing duplicates, two researchers (PJ and SP) independently screened all studies (reviews) by title and abstract. In case of disagreement, two other researchers (OBF and JA) were consulted. The full-text review that followed was performed by PJ and SP; a third researcher (either OBF or JA) would be involved in a tiebreaker situation. Reviews were included if they assessed any psychometric property of instruments measuring the ability to build and maintain social relationships in older persons. Reviews were excluded if they only focused on measuring either the ability to build or the ability to maintain social relationships. Additionally, reviews reporting instruments with a disease-specific focus or measuring older persons’ ability to contribute to society, their family or household were excluded. Reviews were also excluded if the psychometric properties of instruments were not reported separately, the full text was not available, or the study had not been published yet. Reviews were considered if they were written in the English, Spanish and Portuguese languages. We included reviews focusing on older persons aged 60 years and over living in the community or long-term care facilities. The search was not time bounded.

## Results

**Figure 1:**
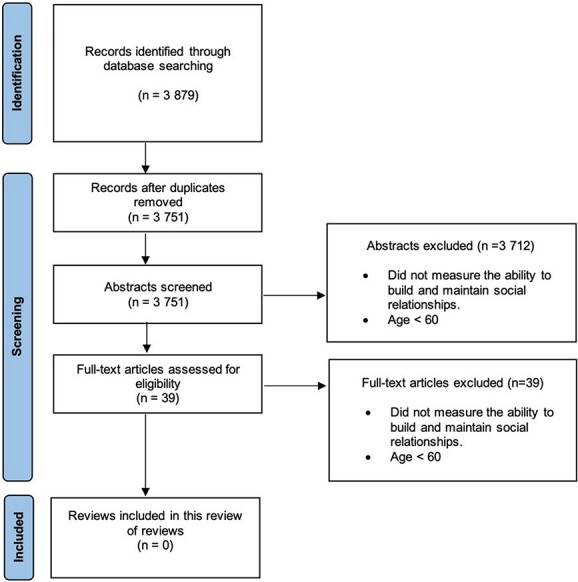
PRISMA flow diagram.

A total of 3,879 reviews were identified, and the titles and abstracts of 3,751 articles were screened after duplicates were removed. After applying exclusion criteria, the full text of 39 articles was screened; this resulted in no articles being included in the qualitative synthesis (Figure 1).

## Discussion

No results were found that fully met the inclusion criteria, thus this study resulted in an empty review. One of the most important factors contributing to this result is the overlap of key constructs (Supplementary Appendix 1) being measured by several of the existing instruments, which leads to current instruments not being suitable for measuring the ability to build and maintain social relationships amongst older persons. To overcome this hindrance, we simplified the search strategy. Notwithstanding, this search retrieved similar results, i.e. after applying the predefined inclusion/exclusion criteria, no reviews would have been included where we could draw instruments measuring the ability to build and maintain social relationships amongst older persons. This result seems to signal a gap in the development of instruments that evaluate simultaneously an older person’s ability to build and maintain social relationships.

To conduct this work, we equated two possible approaches towards identifying instruments that assessed the ability of older persons in building and maintaining social relationships. One possible approach was by conducting a direct assessment of instruments measuring the ability to build and maintain social relationships. The second approach could be by conducting an evaluation by proxy, i.e. researching the ability to build and maintain social relationships by approaching constructs that are more commonly studied amongst current validated instruments in the ageing literature. For this review, we decided on the former approach as we based the review on searching for reviews that had identified instruments that simultaneously assessed the ability to build and maintain social relationships, in line with the five key domains of functional capacity. We recognise that a separate assessment of the ability to build social relationships and the ability to maintain social relationships could have been carried out. This will likely be an essential approach previous to the creation of a new instrument that measures simultaneously the ability of older persons to build and maintain social relationships. For example, in some of the articles selected for full-text reading, instruments with sub-scales that address items necessary to the assessment of the capacity to maintain social relationships were identified, such as:

- Social-family assessment scales (for example, the Barcelona social-familial evaluation scale version [11]). These scales evaluate social relationships, support networks, social interactions and frequency of contacts. However, there seems not to exist a reference to social relationship skills building in these scales.
- Social interaction assessment scales (for example, the Duke Social Support Index [12]). These scales account for the frequency of contacts, support satisfaction and emotional bond. However, there seems not to exist a reference to social relationship skills building in these scales.
- Social network assessment scales (for example, the Lubben Social Network Scale [13]). These scales address the composition and characterisation of social networks, frequency of contacts (neighbours and friends), social support and supportive networks. However, there seems not to exist a reference to social relationship skills building in these scales, too.
- Personality assessment scales (for example, the Big Five Inventory [14]). These scales address the different dimensions of personality that may be useful to assess the ability of older persons to maintain and build social relationships.

Population ageing profoundly impacts our need to understand the ageing process and prepare societies to provide more quality years to their citizens, enhancing agency and the participative and contributive capacity of older citizens. In the context of population ageing, a new approach to models of social relationships for older people is needed. Some attempts have been made in recent years. For example, the Convoy Model and Later-life Family relationships have emphasised the adequacy of the Convoy model to address the challenges of social relations in later life [15]. Another conceptual model for social relations in older persons has highlighted the cross-cutting importance of socio-cultural, social-structural and environmental contexts to individual factors, social relations, and psychosocial and socio-emotional processes; the model also accounts for distal outcomes, such as well-being, health and functioning, social opportunities and social cohesion [16]. One last example is the Differential Investment in Resources Model, which provides a comprehensive and testable framework for understanding changes in social relationships across adulthood into late life, serving as a theoretical basis for establishing how social capital and social function (social exchanges, influence and evaluation) may affect both individual characteristics and living situations [17]. These models assume the need to generate new research hypotheses to understand the underlying biological and ecopsychosocial factors that explain how older persons build and maintain social relations, which supports the need of developing novel instruments that cover both the constructs of building and maintaining social relationships. Measuring both constructs should go hand in hand. One cannot assume that the sole existence of social relationships explains the capacities (or lack thereof) of an older person in building and maintaining social relationships, as this is a complex and multidimensional process.

A new instrument able to measure the ability to build and maintain social relationships as defined by WHO should assess four key dimensions. First, the intrinsic characteristics that allow an older person to be capable of establishing new friendships and relations (e.g. personality traits, physical and mental limitations, ability to use information and communication technologies). Second, the characteristics of an individual’s context (e.g. resources in community settings to facilitate meeting new people, (public) transport networks, secure barrier-free spaces in both rural and urban settings). Third, the type and frequency of social interactions, i.e. measure with whom social interactions occur (e.g. family relatives, long-time friends, neighbours), how they are facilitated (e.g. in-person, via phone or online) and the frequency and duration of these interactions. Lastly, the meaning and satisfaction with the existing relationships, which should capture how older persons experience their social relationships as this can determine their commitment to maintain social contacts and shape their motivation and willingness to expand their network of relationships.

## Conclusion

We argue that the development of an instrument for measuring the ability to build and maintain social relationships in older persons is necessary, as our findings suggest that existing instruments only measure one of the components but not both. Thus, it becomes necessary to assess the ability to maintain social relationships as measured by the frequency, mode and strength of contacts with friends or relatives, in tandem with the assessment of the ability to create relationships. Notably, measuring the ability to create relationships is crucial given that older people lose significant others over the years and this ability becomes essential to enhance their functional capacity. This new instrument should be able to assess: (i) the intrinsic characteristics that allow a person to be capable of establishing new friendships and relations; (ii) the environment in community settings that allow people to meet new people and establish relationships regardless of their age, sex, gender, social economic status or sexual orientation; (iii) type and frequency of social interactions; (iv) meaning and satisfaction of these relationships. Additionally, any new instrument should take into account digital social networks and the role of technology in both building and maintaining social relationships.

## Supplementary Material

aa-23-0441-File002_afad106

## Data Availability

All relevant data are within the paper and its Supplementary Appendixes.

## PROSPERO Registration Number

CRD42022303948.

## References

[1] World Health Organization . World Report on Ageing and Health. Geneva: World Health Organization, 2015.

[2] Kirch W , ed. Functional Ability. Encyclopedia of Public Health. Dordrecht, Netherlands: Springer, 2008; 466.

[3] Durgut O, Gokgun OF, Gencay S. Evaluation of neutrophil-to-lymphocyte ratio, platelet-to-lymphocyte ratio and mean platelet volume in patients with branchial cleft cyst. Indian J Otolaryngol Head Neck Surg 2022; 74: 5465–8. 36742740 10.1007/s12070-021-02789-1 PMC9895673

[4] Zhang X, Dong S. The relationships between social support and loneliness: a meta-analysis and review. Acta Psychol (Amst) 2022; 227: 103616. 35576818 10.1016/j.actpsy.2022.103616

[5] Lynch SA . Who supports whom? How age and gender affect the perceived quality of support from family and friends. Gerontologist 1998; 38: 231–8. 9573668 10.1093/geront/38.2.231

[6] Courtin E, Knapp M. Social isolation, loneliness and health in old age: a scoping review. Health Soc Care Community 2017; 25: 799–812. 26712585 10.1111/hsc.12311

[7] Rapolienė G, Aartsen M. Lonely societies: low trust societies? Further explanations for national variations in loneliness among older Europeans. Eur J Ageing 2022; 19: 485–94. 36052198 10.1007/s10433-021-00649-z PMC9424392

[8] Dang L, Ananthasubramaniam A, Mezuk B. Spotlight on the challenges of depression following retirement and opportunities for interventions. Clin Interv Aging 2022; 17: 1037–56. 35855744 10.2147/CIA.S336301 PMC9288177

[9] Johnson CB, Luther B, Wallace AS, Kulesa MG. Social determinants of health: what are they and how do we screen. Orthop Nurs 2022; 41: 88–100. 35358126 10.1097/NOR.0000000000000829

[10] Ouzzani M, Hammady H, Fedorowicz Z, Elmagarmid A. Rayyan—a web and mobile app for systematic reviews. Syst Rev 2016; 5: 210. 27919275 10.1186/s13643-016-0384-4 PMC5139140

[11] Garcia-Caselles P, Miralles R, Arellano M et al. Validation of a modified version of the Gijon's social-familial evaluation scale (SFES): the "Barcelona SFES version", for patients with cognitive impairment. Arch Gerontol Geriatr Suppl 2004; 38: 201–6. 10.1016/j.archger.2004.04.028 15207415

[12] Koenig HG, Westlund RE, George LK, Hughes DC, Blazer DG, Hybels C. Abbreviating the Duke social support index for use in chronically ill elderly individuals. Psychosomatics 1993; 34: 61–9. 8426892 10.1016/S0033-3182(93)71928-3

[13] Jang Y, Powers DA, Park NS, Chiriboga DA, Chi I, Lubben J. Performance of an abbreviated Lubben social network scale (LSNS-6) in three ethnic groups of older Asian Americans. Gerontologist 2022; 62: e73–81. 33021635 10.1093/geront/gnaa156 PMC13377234

[14] John OP, Srivastava S. The Big Five Trait Taxonomy: History, Measurement, and Theoretical Perspectives. In: Pervin LA, John OP, eds. Handbook of Personality: Theory and Research. New York: Guilford Press, 1999; 102–38.

[15] Fuller HR, Ajrouch KJ, Antonucci TC. The convoy model and later-life family relationships. J Fam Theory Rev 2020; 12: 126–46. 32536976 10.1111/jftr.12376 PMC7283809

[16] Burholt V, Winter B, Aartsen M et al. A critical review and development of a conceptual model of exclusion from social relations for older people. Eur J Ageing 2020; 17: 3–19. 32158368 10.1007/s10433-019-00506-0 PMC7040153

[17] Huxhold O, Fiori KL, Windsor T. Rethinking social relationships in adulthood: the differential Investment of Resources Model. Pers Soc Psychol Rev 2022; 26: 57–82. 35001730 10.1177/10888683211067035 PMC8978474

