# Supplementary material for: A systematic review of reviews on the psychometric properties of measures of older persons’ ability to build and maintain social relationships
Source: Age Ageing. 2023 Oct 30;52(Suppl 4):iv133–7. doi: 10.1093/ageing/afad106 (PMC10615056; doi:10.1093/ageing/afad106)
Supplement: aa-23-0441-File002_afad106 [file aa-23-0441-file002_afad106.docx]

World Health Organisation: *Measurements of Healthy Ageing*

**A systematic review of reviews on the psychometric properties of measures of older persons’ ability to build and maintain social relationships**

**SUPPLEMENTARY DATA**

- **Appendix 1 –** Key terms and definitions
- **Appendix 2 –** Search strategy

**Appendix 1 –** Key terms and definitions

| Key term | Definition |
| --- | --- |
| Loneliness | A subjective unpleasant or distressing feeling of a lack of connection to other people, along with a desire for more, or more satisfying, social relationships^1^, results from a discrepancy between desired and actual social connections^2^. |
| Social connection | Having a variety of relationships (from close personal ties, such as family and friends, to weaker ties, such as acquaintances and strangers); relationships one can rely upon for support; and relationships that are trusted, high quality, and satisfying. These relationships reflect a multitude of influences, including the diversity of our individual cultures and experiences and one’s biology ^1^. |
| Social interaction | Any process that involves reciprocal stimulation or response between two or more individuals. These can range from the first encounters between parent and offspring to complex interactions with multiple individuals in adult life. Social interaction includes the development of cooperation and competition, the influence of status and social roles, and the dynamics of group behavior, leadership, and conformity. Persistent social interaction between specific individuals leads to the formation of [social relationships](https://dictionary.apa.org/social-relationships). It is only through close observation of social interaction that [social organization](https://dictionary.apa.org/social-organization) and [social structure](https://dictionary.apa.org/social-structure) can be inferred^3^. |
| Social network | The relatively organized set of relationships that an individual or group has with others, including types and methods of communication, patterns of liking and disliking, and the strength of interpersonal connections. Such structures may be analysed quantitatively using [social network analysis](https://dictionary.apa.org/social-network-analysis)^3^. |
| Social relationship | The sum of the [social interactions](https://dictionary.apa.org/social-interactions) between individuals over a period of time. Momentary social interactions can be described in terms of parental care, dominant–subordinate or aggressive–fearful interactions, and so on, but a social relationship is the emergent quality from repeated interactions. A [dyad](https://dictionary.apa.org/dyad) (interacting pair) may have a generally positive or generally negative social relationship that is reciprocal or complementary. Dyads with long-term social relationships will adjust behaviour with each other according to feedback received^3^. |
| Social support | The provision of assistance or comfort to others, typically to help them cope with biological, psychological, and social stressors. Support may arise from any interpersonal relationship in an individual’s social network, involving family members, friends, neighbours, religious institutions, colleagues, caregivers, or [support groups](https://dictionary.apa.org/support-groups). It may take the form of practical help (e.g., doing chores, offering advice), tangible support that involves giving money or other direct material assistance, and emotional support that allows the individual to feel valued, accepted, and understood. See also [coping](https://dictionary.apa.org/coping); [invisible support](https://dictionary.apa.org/invisible-support)^3^. |
| Social ties | All persons for which the ego has: (a) subjective awareness; (b) a mental representation of how they relate; and (c) a belief in their mutual influence on each other ^4^. |
| Social isolation | The objective state of having a small network of kin and non-kin relationships and thus few or infrequent interactions with others^2^. Refers to having objectively few social relationships, social roles, group memberships, and infrequent social interaction^1^. |

1 – Global Initiative on Loneliness and Connection. Position Statements on Addressing Social Isolation, Loneliness, and the Power of Human Connection. 2022. Online at <https://www.gilc.global/_files/ugd/410bdf_74fffc2d18984b0e8217288b1b12d199.pdf>. Consulted on 30/01/2023.

2 – WHO. Social isolation and loneliness among older people: advocacy brief. Geneva: World Health Organization; 2021.

3 – APA Dictionary of Psychology. Online at <https://dictionary.apa.org/>. Consulted on 30/01/2023.

4 – Huxhold, O., Fiori, K. L., & Windsor, T. (2022). Rethinking Social Relationships in Adulthood: The Differential Investment of Resources Model. Personality and Social Psychology Review, 26(1), 57–82. https://doi.org/10.1177/10888683211067035

**Appendix 2 –** Search strategy

((MH "Psychometrics") OR (MH "Measurement Issues and Assessments") OR (MH "Validity") OR (MH "Predictive Validity") OR (MH "Reliability and Validity") OR (MH "Internal Validity") OR (MH "Face Validity") OR (MH "External Validity") OR (MH "Discriminant Validity") OR (MH "Criterion-Related Validity") OR (MH "Consensual Validity") OR (MH "Concurrent Validity") OR (MH "Qualitative Validity") OR (MH "Construct Validity") OR (MH "Content Validity") OR (MH "Questionnaire Validation") OR (MH "Validation Studies") OR (MH "Test-Retest Reliability") OR (MH "Sensitivity and Specificity") OR (MH "Reproducibility of Results") OR (MH "Reliability") OR (MH "Interrater Reliability") OR (MH "Interrater Reliability") OR (MH "Measurement Error") OR (MH "Bias (Research)") OR (MH "Selection Bias") OR (MH "Sampling Bias") OR (MH "Precision") OR (MH "Sample Size Determination") OR (MH "Repeated Measures") OR (Psychometric* or Reliability or Validity* or Reproducibility or Bias) AND ((exp Aged/ OR (Senior$1 or Elderly or Older).tw. ) OR (MH " Frail Elderly") OR (Exp Aged/ or Middle Aged/) OR ((Old* or Age*) adj3 (People* or Person* or Adult* or Women* or Men* or Citizen* or Resident*)).tw. OR (Pension* or Retire* or Elderly or Senior* or Geriatric*).tw.) AND ((MH “Social Contact”) OR (MH “Social Assessment”) OR (MH “Social Network”) OR (MH “Social Support”) OR (MH “Social Scales”) OR (MH “Interpersonal relationships”))

Note: The search was adapted to each search engine.
